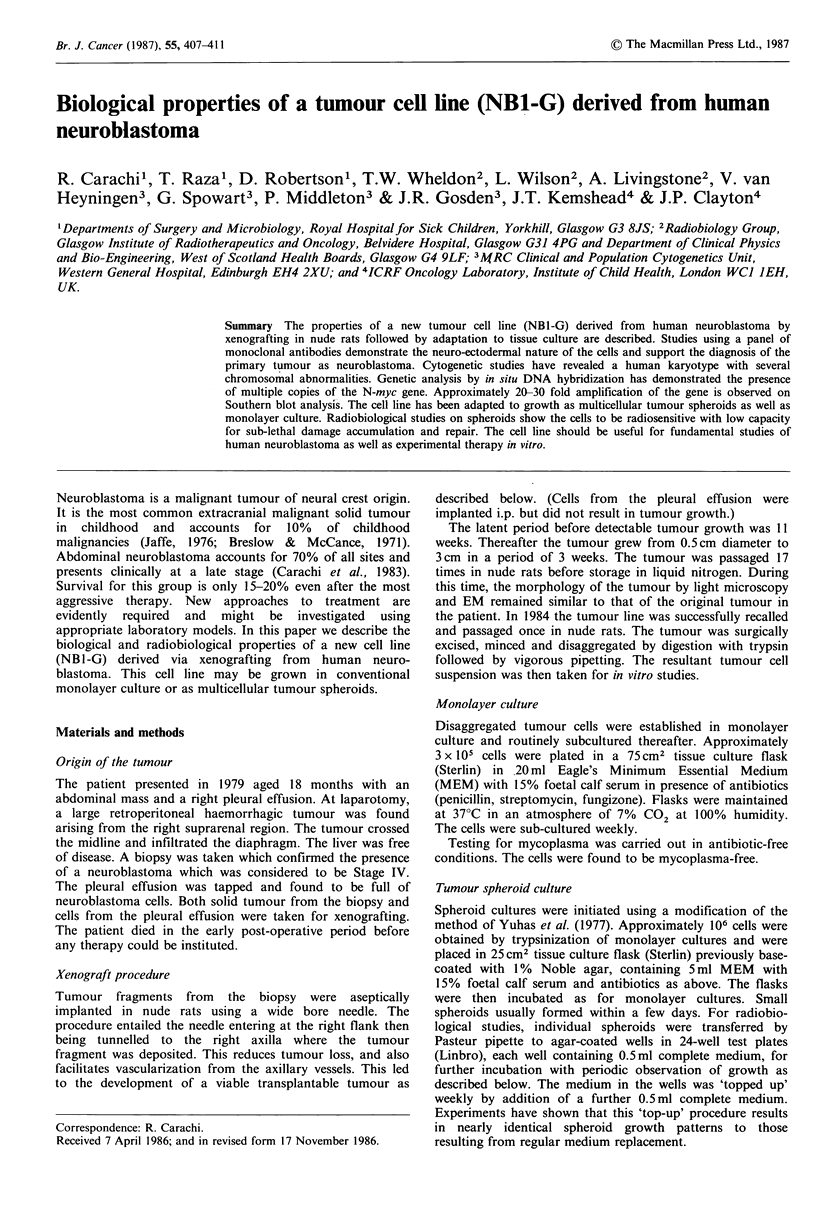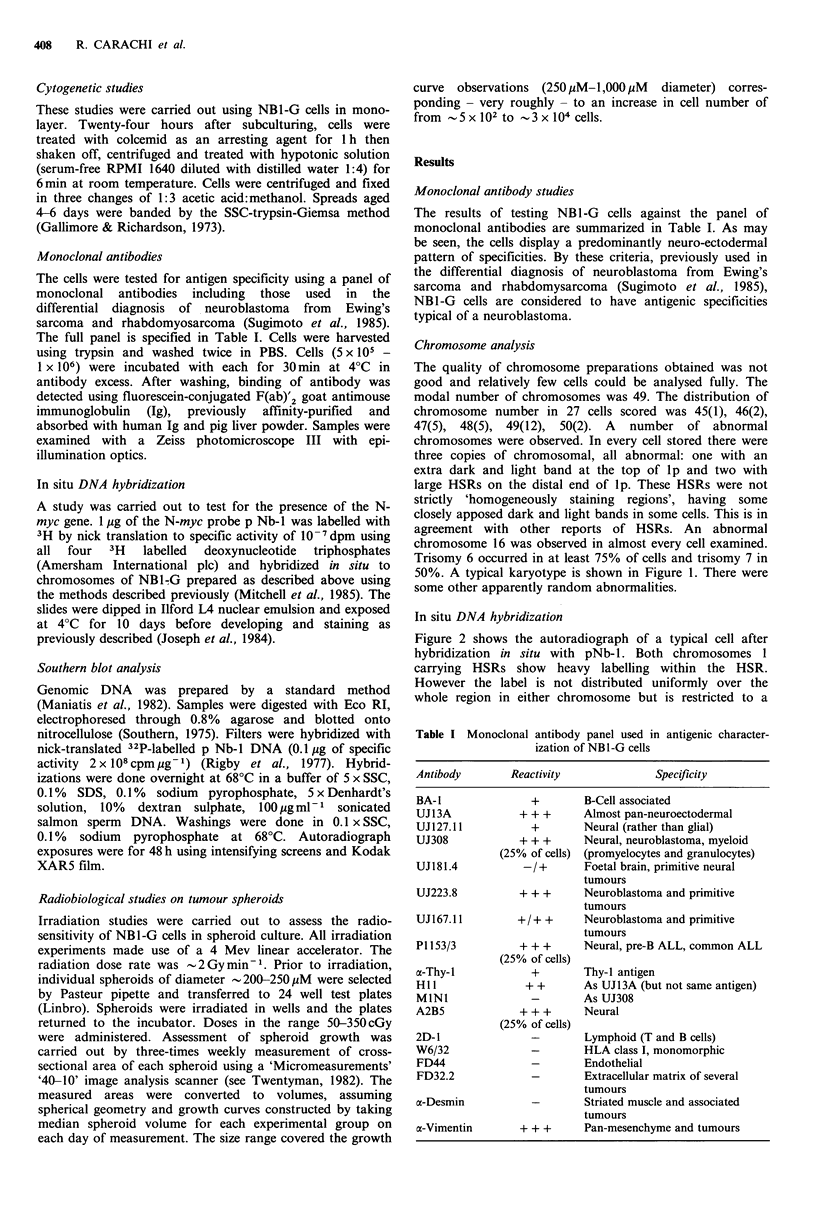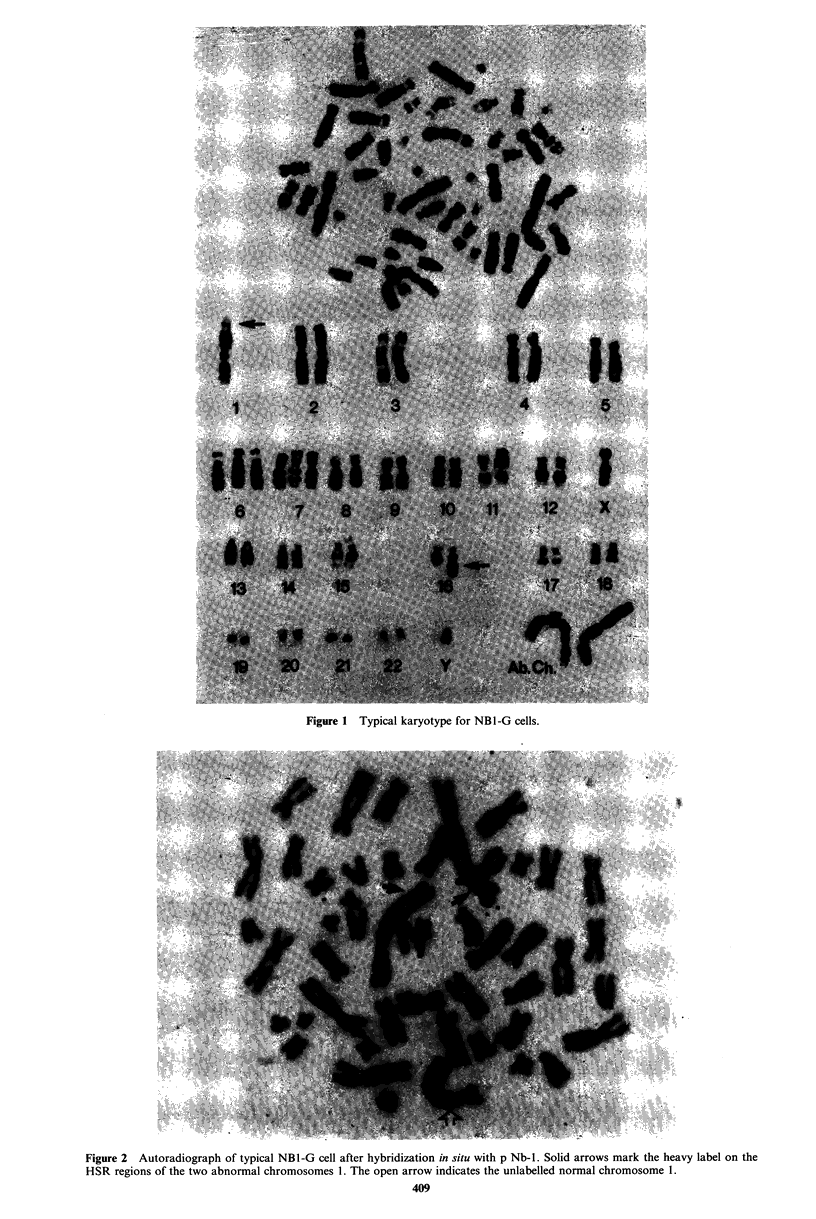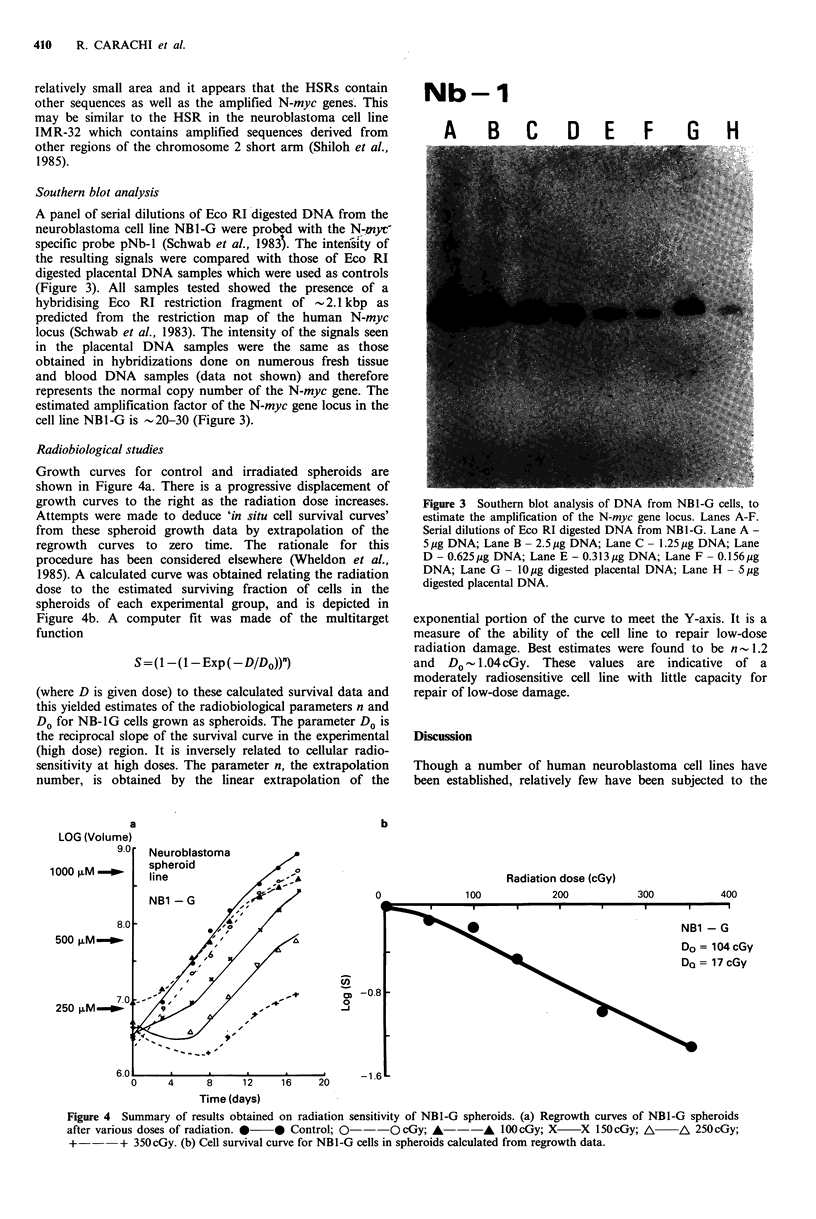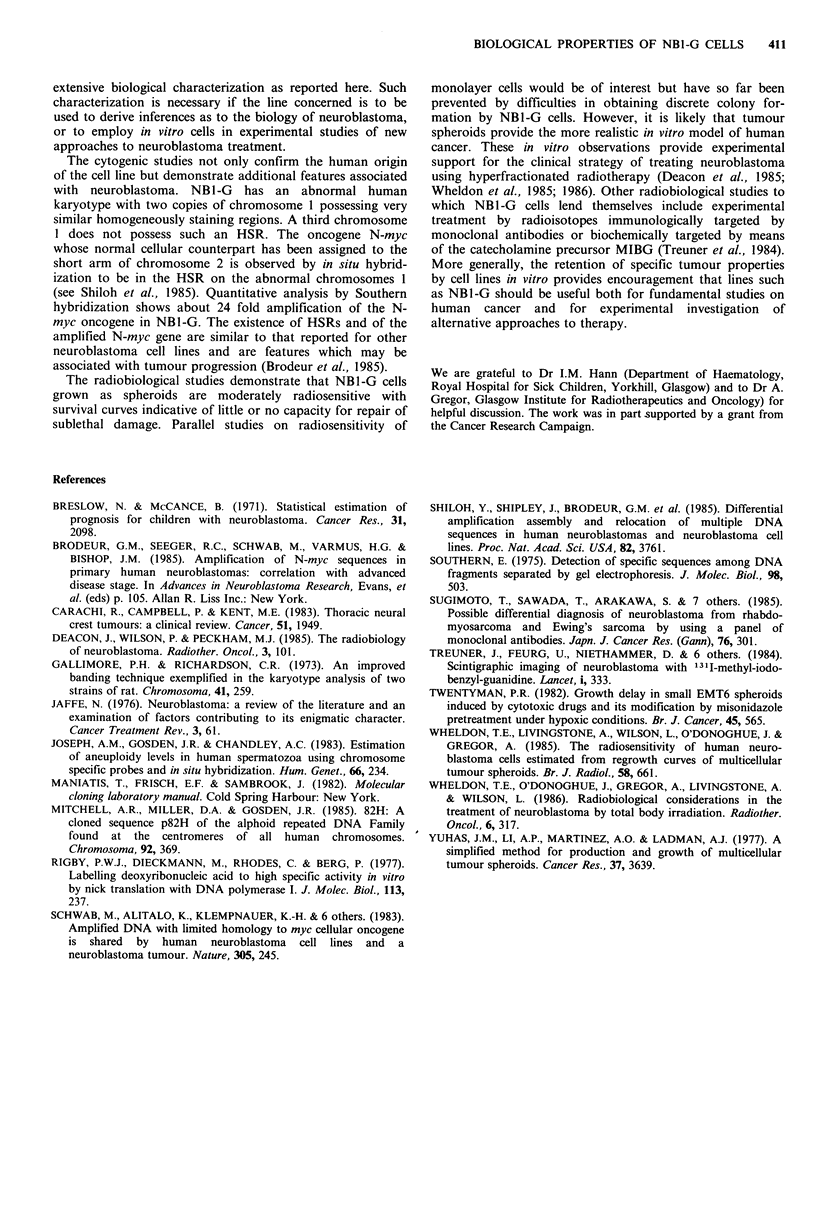# Biological properties of a tumour cell line (NB1-G) derived from human neuroblastoma.

**DOI:** 10.1038/bjc.1987.80

**Published:** 1987-04

**Authors:** R. Carachi, T. Raza, D. Robertson, T. W. Wheldon, L. Wilson, A. Livingstone, V. van Heyningen, G. Spowart, P. Middleton, J. R. Gosden

## Abstract

**Images:**


*Br. J. Cancer* (1987), 55, 407–411 © The Macmillan Press Ltd., 1987

### Biological properties of a tumour cell line (NB1-G) derived from human neuroblastoma

R. Carachi[1], T. Raza[1], D. Robertson[1], T.W. Wheldon[2], L. Wilson[2], A. Livingstone[2], V. van Heyningen[3], G. Spowart[3], P. Middleton[3] & J.R. Gosden[3], J.T. Kemshead[4] & J.P. Clayton[4]

[1]*Departments of Surgery and Microbiology, Royal Hospital for Sick Children, Yorkhill, Glasgow G3 8JS;* [2]*Radiobiology Group, Glasgow Institute of Radiotherapeutics and Oncology, Belvidere Hospital, Glasgow G31 4PG and Department of Clinical Physics and Bio-Engineering, West of Scotland Health Boards, Glasgow G4 9LF;* [3]*MRC Clinical and Population Cytogenetics Unit, Western General Hospital, Edinburgh EH4 2XU; and* [4]*ICRF Oncology Laboratory, Institute of Child Health, London WC1 1EH, UK.*

**Summary** The properties of a new tumour cell line (NB1-G) derived from human neuroblastoma by xenografting in nude rats followed by adaptation to tissue culture are described. Studies using a panel of monoclonal antibodies demonstrate the neuro-ectodermal nature of the cells and support the diagnosis of the primary tumour as neuroblastoma. Cytogenetic studies have revealed a human karyotype with several chromosomal abnormalities. Genetic analysis by *in situ* DNA hybridization has demonstrated the presence of multiple copies of the N-*myc* gene. Approximately 20–30 fold amplification of the gene is observed on Southern blot analysis. The cell line has been adapted to growth as multicellular tumour spheroids as well as monolayer culture. Radiobiological studies on spheroids show the cells to be radiosensitive with low capacity for sub-lethal damage accumulation and repair. The cell line should be useful for fundamental studies of human neuroblastoma as well as experimental therapy *in vitro*.

Neuroblastoma is a malignant tumour of neural crest origin. It is the most common extracranial malignant solid tumour in childhood and accounts for 10% of childhood malignancies (Jaffe, 1976; Breslow & McCance, 1971). Abdominal neuroblastoma accounts for 70% of all sites and presents clinically at a late stage (Carachi *et al.*, 1983). Survival for this group is only 15–20% even after the most aggressive therapy. New approaches to treatment are evidently required and might be investigated using appropriate laboratory models. In this paper we describe the biological and radiobiological properties of a new cell line (NB1-G) derived via xenografting from human neuroblastoma. This cell line may be grown in conventional monolayer culture or as multicellular tumour spheroids.

### Materials and methods

### *Origin of the tumour*

The patient presented in 1979 aged 18 months with an abdominal mass and a right pleural effusion. At laparotomy, a large retroperitoneal haemorrhagic tumour was found arising from the right suprarenal region. The tumour crossed the midline and infiltrated the diaphragm. The liver was free of disease. A biopsy was taken which confirmed the presence of a neuroblastoma which was considered to be Stage IV. The pleural effusion was tapped and found to be full of neuroblastoma cells. Both solid tumour from the biopsy and cells from the pleural effusion were taken for xenografting. The patient died in the early post-operative period before any therapy could be instituted.

### *Xenograft procedure*

Tumour fragments from the biopsy were aseptically implanted in nude rats using a wide bore needle. The procedure entailed the needle entering at the right flank then being tunnelled to the right axilla where the tumour fragment was deposited. This reduces tumour loss, and also facilitates vascularization from the axillary vessels. This led to the development of a viable transplantable tumour as described below. (Cells from the pleural effusion were implanted i.p. but did not result in tumour growth.)

The latent period before detectable tumour growth was 11 weeks. Thereafter the tumour grew from 0.5 cm diameter to 3 cm in a period of 3 weeks. The tumour was passaged 17 times in nude rats before storage in liquid nitrogen. During this time, the morphology of the tumour by light microscopy and EM remained similar to that of the original tumour in the patient. In 1984 the tumour line was successfully recalled and passaged once in nude rats. The tumour was surgically excised, minced and disaggregated by digestion with trypsin followed by vigorous pipetting. The resultant tumour cell suspension was then taken for *in vitro* studies.

### *Monolayer culture*

Disaggregated tumour cells were established in monolayer culture and routinely subcultured thereafter. Approximately $3 \times 10^5$ cells were plated in a 75 cm$^2$ tissue culture flask (Sterlin) in 20 ml Eagle's Minimum Essential Medium (MEM) with 15% foetal calf serum in presence of antibiotics (penicillin, streptomycin, fungizone). Flasks were maintained at 37°C in an atmosphere of 7% $CO_2$ at 100% humidity. The cells were sub-cultured weekly.

Testing for mycoplasma was carried out in antibiotic-free conditions. The cells were found to be mycoplasma-free.

### *Tumour spheroid culture*

Spheroid cultures were initiated using a modification of the method of Yuhas *et al.* (1977). Approximately $10^6$ cells were obtained by trypsinization of monolayer cultures and were placed in 25 cm$^2$ tissue culture flask (Sterlin) previously base-coated with 1% Noble agar, containing 5 ml MEM with 15% foetal calf serum and antibiotics as above. The flasks were then incubated as for monolayer cultures. Small spheroids usually formed within a few days. For radiobiological studies, individual spheroids were transferred by Pasteur pipette to agar-coated wells in 24-well test plates (Linbro), each well containing 0.5 ml complete medium, for further incubation with periodic observation of growth as described below. The medium in the wells was 'topped up' weekly by addition of a further 0.5 ml complete medium. Experiments have shown that this 'top-up' procedure results in nearly identical spheroid growth patterns to those resulting from regular medium replacement.

Correspondence: R. Carachi.
Received 7 April 1986; and in revised form 17 November 1986.

*Cytogenetic studies*

These studies were carried out using NB1-G cells in monolayer. Twenty-four hours after subculturing, cells were treated with colcemid as an arresting agent for 1 h then shaken off, centrifuged and treated with hypotonic solution (serum-free RPMI 1640 diluted with distilled water 1:4) for 6 min at room temperature. Cells were centrifuged and fixed in three changes of 1:3 acetic acid:methanol. Spreads aged 4–6 days were banded by the SSC-trypsin-Giemsa method (Gallimore & Richardson, 1973).

*Monoclonal antibodies*

The cells were tested for antigen specificity using a panel of monoclonal antibodies including those used in the differential diagnosis of neuroblastoma from Ewing's sarcoma and rhabdomyosarcoma (Sugimoto *et al.*, 1985). The full panel is specified in Table I. Cells were harvested using trypsin and washed twice in PBS. Cells ($5\times10^5$ – $1\times10^6$) were incubated with each for 30 min at 4°C in antibody excess. After washing, binding of antibody was detected using fluorescein-conjugated $F(ab)'_2$ goat antimouse immunoglobulin (Ig), previously affinity-purified and absorbed with human Ig and pig liver powder. Samples were examined with a Zeiss photomicroscope III with epi-illumination optics.

In situ *DNA hybridization*

A study was carried out to test for the presence of the N-*myc* gene. 1 $\mu$g of the N-*myc* probe p Nb-1 was labelled with $^3H$ by nick translation to specific activity of $10^{-7}$ dpm using all four $^3H$ labelled deoxynucleotide triphosphates (Amersham International plc) and hybridized *in situ* to chromosomes of NB1-G prepared as described above using the methods described previously (Mitchell *et al.*, 1985). The slides were dipped in Ilford L4 nuclear emulsion and exposed at 4°C for 10 days before developing and staining as previously described (Joseph *et al.*, 1984).

*Southern blot analysis*

Genomic DNA was prepared by a standard method (Maniatis *et al.*, 1982). Samples were digested with Eco RI, electrophoresed through 0.8% agarose and blotted onto nitrocellulose (Southern, 1975). Filters were hybridized with nick-translated $^{32}P$-labelled p Nb-1 DNA (0.1 $\mu$g of specific activity $2\times10^8$ cpm $\mu g^{-1}$) (Rigby *et al.*, 1977). Hybridizations were done overnight at 68°C in a buffer of 5 × SSC, 0.1% SDS, 0.1% sodium pyrophosphate, 5 × Denhardt's solution, 10% dextran sulphate, 100 $\mu$g ml$^{-1}$ sonicated salmon sperm DNA. Washings were done in 0.1 × SSC, 0.1% sodium pyrophosphate at 68°C. Autoradiograph exposures were for 48 h using intensifying screens and Kodak XAR5 film.

*Radiobiological studies on tumour spheroids*

Irradiation studies were carried out to assess the radiosensitivity of NB1-G cells in spheroid culture. All irradiation experiments made use of a 4 Mev linear accelerator. The radiation dose rate was $\sim$2 Gy min$^{-1}$. Prior to irradiation, individual spheroids of diameter $\sim$200–250 $\mu$M were selected by Pasteur pipette and transferred to 24 well test plates (Linbro). Spheroids were irradiated in wells and the plates returned to the incubator. Doses in the range 50–350 cGy were administered. Assessment of spheroid growth was carried out by three-times weekly measurement of cross-sectional area of each spheroid using a 'Micromeasurements' '40–10' image analysis scanner (see Twentyman, 1982). The measured areas were converted to volumes, assuming spherical geometry and growth curves constructed by taking median spheroid volume for each experimental group on each day of measurement. The size range covered the growth curve observations (250 $\mu$M–1,000 $\mu$M diameter) corresponding – very roughly – to an increase in cell number of from $\sim5\times10^2$ to $\sim3\times10^4$ cells.

### Results

*Monoclonal antibody studies*

The results of testing NB1-G cells against the panel of monoclonal antibodies are summarized in Table I. As may be seen, the cells display a predominantly neuro-ectodermal pattern of specificities. By these criteria, previously used in the differential diagnosis of neuroblastoma from Ewing's sarcoma and rhabdomysarcoma (Sugimoto *et al.*, 1985), NB1-G cells are considered to have antigenic specificities typical of a neuroblastoma.

*Chromosome analysis*

The quality of chromosome preparations obtained was not good and relatively few cells could be analysed fully. The modal number of chromosomes was 49. The distribution of chromosome number in 27 cells scored was 45(1), 46(2), 47(5), 48(5), 49(12), 50(2). A number of abnormal chromosomes were observed. In every cell stored there were three copies of chromosomal, all abnormal: one with an extra dark and light band at the top of 1p and two with large HSRs on the distal end of 1p. These HSRs were not strictly 'homogeneously staining regions', having some closely apposed dark and light bands in some cells. This is in agreement with other reports of HSRs. An abnormal chromosome 16 was observed in almost every cell examined. Trisomy 6 occurred in at least 75% of cells and trisomy 7 in 50%. A typical karyotype is shown in Figure 1. There were some other apparently random abnormalities.

In situ *DNA hybridization*

Figure 2 shows the autoradiograph of a typical cell after hybridization *in situ* with pNb-1. Both chromosomes 1 carrying HSRs show heavy labelling within the HSR. However the label is not distributed uniformly over the whole region in either chromosome but is restricted to a

**Table I** Monoclonal antibody panel used in antigenic characterization of NB1-G cells

| *Antibody* | *Reactivity* | *Specificity* |
|---|---|---|
| BA-1 | + | B-Cell associated |
| UJ13A | + + + | Almost pan-neuroectodermal |
| UJ127.11 | + | Neural (rather than glial) |
| UJ308 | + + + (25% of cells) | Neural, neuroblastoma, myeloid (promyelocytes and granulocytes) |
| UJ181.4 | −/+ | Foetal brain, primitive neural tumours |
| UJ223.8 | + + + | Neuroblastoma and primitive tumours |
| UJ167.11 | +/+ + | Neuroblastoma and primitive tumours |
| P1153/3 | + + + (25% of cells) | Neural, pre-B ALL, common ALL |
| α-Thy-1 | + | Thy-1 antigen |
| H11 | + + | As UJ13A (but not same antigen) |
| M1N1 | − | As UJ308 |
| A2B5 | + + + (25% of cells) | Neural |
| 2D-1 | − | Lymphoid (T and B cells) |
| W6/32 | − | HLA class I, monomorphic |
| FD44 | − | Endothelial |
| FD32.2 | − | Extracellular matrix of several tumours |
| α-Desmin | − | Striated muscle and associated tumours |
| α-Vimentin | + + + | Pan-mesenchyme and tumours |

**Figure 1** Typical karyotype for NB1-G cells.

**Figure 2** Autoradiograph of typical NB1-G cell after hybridization *in situ* with p Nb-1. Solid arrows mark the heavy label on the HSR regions of the two abnormal chromosomes 1. The open arrow indicates the unlabelled normal chromosome 1.

relatively small area and it appears that the HSRs contain other sequences as well as the amplified N-*myc* genes. This may be similar to the HSR in the neuroblastoma cell line IMR-32 which contains amplified sequences derived from other regions of the chromosome 2 short arm (Shiloh *et al.*, 1985).

*Southern blot analysis*

A panel of serial dilutions of Eco RI digested DNA from the neuroblastoma cell line NB1-G were probed with the N-*myc* specific probe pNb-1 (Schwab *et al.*, 1983). The intensity of the resulting signals were compared with those of Eco RI digested placental DNA samples which were used as controls (Figure 3). All samples tested showed the presence of a hybridising Eco RI restriction fragment of ~2.1 kbp as predicted from the restriction map of the human N-*myc* locus (Schwab *et al.*, 1983). The intensity of the signals seen in the placental DNA samples were the same as those obtained in hybridizations done on numerous fresh tissue and blood DNA samples (data not shown) and therefore represents the normal copy number of the N-*myc* gene. The estimated amplification factor of the N-*myc* gene locus in the cell line NB1-G is ~20–30 (Figure 3).

*Radiobiological studies*

Growth curves for control and irradiated spheroids are shown in Figure 4a. There is a progressive displacement of growth curves to the right as the radiation dose increases. Attempts were made to deduce '*in situ* cell survival curves' from these spheroid growth data by extrapolation of the regrowth curves to zero time. The rationale for this procedure has been considered elsewhere (Wheldon *et al.*, 1985). A calculated curve was obtained relating the radiation dose to the estimated surviving fraction of cells in the spheroids of each experimental group, and is depicted in Figure 4b. A computer fit was made of the multitarget function

$$S=(1-(1-\mathrm{Exp}(-D/D_0))^n)$$

(where $D$ is given dose) to these calculated survival data and this yielded estimates of the radiobiological parameters $n$ and $D_0$ for NB-1G cells grown as spheroids. The parameter $D_0$ is the reciprocal slope of the survival curve in the experimental (high dose) region. It is inversely related to cellular radiosensitivity at high doses. The parameter $n$, the extrapolation number, is obtained by the linear extrapolation of the exponential portion of the curve to meet the Y-axis. It is a measure of the ability of the cell line to repair low-dose radiation damage. Best estimates were found to be $n \sim 1.2$ and $D_0 \sim 1.04$ cGy. These values are indicative of a moderately radiosensitive cell line with little capacity for repair of low-dose damage.

**Figure 3** Southern blot analysis of DNA from NB1-G cells, to estimate the amplification of the N-*myc* gene locus. Lanes A-F. Serial dilutions of Eco RI digested DNA from NB1-G. Lane A – 5 μg DNA; Lane B – 2.5 μg DNA; Lane C – 1.25 μg DNA; Lane D – 0.625 μg DNA; Lane E – 0.313 μg DNA; Lane F – 0.156 μg DNA; Lane G – 10 μg digested placental DNA; Lane H – 5 μg digested placental DNA.

### Discussion

Though a number of human neuroblastoma cell lines have been established, relatively few have been subjected to the

**Figure 4** Summary of results obtained on radiation sensitivity of NB1-G spheroids. (a) Regrowth curves of NB1-G spheroids after various doses of radiation. ●——● Control; O———O cGy; ▲———▲ 100 cGy; X——X 150 cGy; △——△ 250 cGy; +———+ 350 cGy. (b) Cell survival curve for NB1-G cells in spheroids calculated from regrowth data.

extensive biological characterization as reported here. Such characterization is necessary if the line concerned is to be used to derive inferences as to the biology of neuroblastoma, or to employ *in vitro* cells in experimental studies of new approaches to neuroblastoma treatment.

The cytogenic studies not only confirm the human origin of the cell line but demonstrate additional features associated with neuroblastoma. NB1-G has an abnormal human karyotype with two copies of chromosome 1 possessing very similar homogeneously staining regions. A third chromosome 1 does not possess such an HSR. The oncogene N-*myc* whose normal cellular counterpart has been assigned to the short arm of chromosome 2 is observed by *in situ* hybridization to be in the HSR on the abnormal chromosomes 1 (see Shiloh *et al.*, 1985). Quantitative analysis by Southern hybridization shows about 24 fold amplification of the N-*myc* oncogene in NB1-G. The existence of HSRs and of the amplified N-*myc* gene are similar to that reported for other neuroblastoma cell lines and are features which may be associated with tumour progression (Brodeur *et al.*, 1985).

The radiobiological studies demonstrate that NB1-G cells grown as spheroids are moderately radiosensitive with survival curves indicative of little or no capacity for repair of sublethal damage. Parallel studies on radiosensitivity of monolayer cells would be of interest but have so far been prevented by difficulties in obtaining discrete colony formation by NB1-G cells. However, it is likely that tumour spheroids provide the more realistic *in vitro* model of human cancer. These *in vitro* observations provide experimental support for the clinical strategy of treating neuroblastoma using hyperfractionated radiotherapy (Deacon *et al.*, 1985; Wheldon *et al.*, 1985; 1986). Other radiobiological studies to which NB1-G cells lend themselves include experimental treatment by radioisotopes immunologically targeted by monoclonal antibodies or biochemically targeted by means of the catecholamine precursor MIBG (Treuner *et al.*, 1984). More generally, the retention of specific tumour properties by cell lines *in vitro* provides encouragement that lines such as NB1-G should be useful both for fundamental studies on human cancer and for experimental investigation of alternative approaches to therapy.

We are grateful to Dr I.M. Hann (Department of Haematology, Royal Hospital for Sick Children, Yorkhill, Glasgow) and to Dr A. Gregor, Glasgow Institute for Radiotherapeutics and Oncology) for helpful discussion. The work was in part supported by a grant from the Cancer Research Campaign.

### References

BRESLOW, N. & MCCANCE, B. (1971). Statistical estimation of prognosis for children with neuroblastoma. *Cancer Res.*, **31**, 2098.

BRODEUR, G.M., SEEGER, R.C., SCHWAB, M., VARMUS, H.G. & BISHOP, J.M. (1985). Amplification of N-*myc* sequences in primary human neuroblastomas: correlation with advanced disease stage. In *Advances in Neuroblastoma Research*, Evans, *et al.* (eds) p. 105. Allan R. Liss Inc.: New York.

CARACHI, R., CAMPBELL, P. & KENT, M.E. (1983). Thoracic neural crest tumours: a clinical review. *Cancer*, **51**, 1949.

DEACON, J., WILSON, P. & PECKHAM, M.J. (1985). The radiobiology of neuroblastoma. *Radiother. Oncol.*, **3**, 101.

GALLIMORE, P.H. & RICHARDSON, C.R. (1973). An improved banding technique exemplified in the karyotype analysis of two strains of rat. *Chromosoma*, **41**, 259.

JAFFE, N. (1976). Neuroblastoma: a review of the literature and an examination of factors contributing to its enigmatic character. *Cancer Treatment Rev.*, **3**, 61.

JOSEPH, A.M., GOSDEN, J.R. & CHANDLEY, A.C. (1983). Estimation of aneuploidy levels in human spermatozoa using chromosome specific probes and *in situ* hybridization. *Hum. Genet.*, **66**, 234.

MANIATIS, T., FRISCH, E.F. & SAMBROOK, J. (1982). *Molecular cloning laboratory manual.* Cold Spring Harbour: New York.

MITCHELL, A.R., MILLER, D.A. & GOSDEN, J.R. (1985). 82H: A cloned sequence p82H of the alphoid repeated DNA Family found at the centromeres of all human chromosomes. *Chromosoma*, **92**, 369.

RIGBY, P.W.J., DIECKMANN, M., RHODES, C. & BERG, P. (1977). Labelling deoxyribonucleic acid to high specific activity *in vitro* by nick translation with DNA polymerase I. *J. Molec. Biol.*, **113**, 237.

SCHWAB, M., ALITALO, K., KLEMPNAUER, K.-H. & 6 others. (1983). Amplified DNA with limited homology to *myc* cellular oncogene is shared by human neuroblastoma cell lines and a neuroblastoma tumour. *Nature*, **305**, 245.

SHILOH, Y., SHIPLEY, J., BRODEUR, G.M. *et al.* (1985). Differential amplification assembly and relocation of multiple DNA sequences in human neuroblastomas and neuroblastoma cell lines. *Proc. Nat. Acad. Sci. USA*, **82**, 3761.

SOUTHERN, E. (1975). Detection of specific sequences among DNA fragments separated by gel electrophoresis. *J. Molec. Biol.*, **98**, 503.

SUGIMOTO, T., SAWADA, T., ARAKAWA, S. & 7 others. (1985). Possible differential diagnosis of neuroblastoma from rhabdomyosarcoma and Ewing's sarcoma by using a panel of monoclonal antibodies. *Japn. J. Cancer Res.* (*Gann*), **76**, 301.

TREUNER, J., FEURG, U., NIETHAMMER, D. & 6 others. (1984). Scintigraphic imaging of neuroblastoma with $^{131}$I-methyl-iodo-benzyl-guanidine. *Lancet*, **i**, 333.

TWENTYMAN, P.R. (1982). Growth delay in small EMT6 spheroids induced by cytotoxic drugs and its modification by misonidazole pretreatment under hypoxic conditions. *Br. J. Cancer*, **45**, 565.

WHELDON, T.E., LIVINGSTONE, A., WILSON, L., O'DONOGHUE, J. & GREGOR, A. (1985). The radiosensitivity of human neuroblastoma cells estimated from regrowth curves of multicellular tumour spheroids. *Br. J. Radiol.*, **58**, 661.

WHELDON, T.E., O'DONOGHUE, J., GREGOR, A., LIVINGSTONE, A. & WILSON, L. (1986). Radiobiological considerations in the treatment of neuroblastoma by total body irradiation. *Radiother. Oncol.*, **6**, 317.

YUHAS, J.M., LI, A.P., MARTINEZ, A.O. & LADMAN, A.J. (1977). A simplified method for production and growth of multicellular tumour spheroids. *Cancer Res.*, **37**, 3639.